# Couples extrinsic emotion regulation questionnaire: Psychometric validation in a Chilean population

**DOI:** 10.1371/journal.pone.0252329

**Published:** 2021-06-02

**Authors:** Ana Kinkead, Susana Sanduvete-Chaves, Salvador Chacón-Moscoso, Christian E. Salas

**Affiliations:** 1 Directorate of Research, Vice-rector for Research and Doctorates, Universidad Autónoma de Chile, Santiago, Metropolitan Region, Chile; 2 Doctorate in Psychology, Faculty of Psychology, Universidad Diego Portales, Santiago, Metropolitan Region, Chile; 3 Departamento de Psicología Experimental, Facultad de Psicología, Universidad de Sevilla, Seville, Spain; 4 Departamento de Psicología, Universidad Autónoma de Chile, Santiago, Chile; 5 Faculty of Psychology, Universidad Diego Portales, Santiago, Metropolitan Region, Chile; Aalborg University, DENMARK

## Abstract

The way couples regulate their emotions affects the quality of their relationship. Despite this, no evidence-based scales of validity and reliability can measure the intention to regulate emotions in the romantic dyad. In order to address this gap, we developed the Couples Extrinsic Emotion Regulation (CEER) questionnaire. First, we adapted the “Others” subscale from the Emotion Regulation of Others and Self questionnaire (EROS) for any close relationship to measure the intention to regulate emotions in couples; second, the psychometric properties of the CEER questionnaire were studied. For the content validity assessment, 23 experts (47.8% of whom worked in social and health psychology and the psychology of emotions, 17.4% in couples’ therapy, and 34.8% in social science methodologies) participated. A total of 528 Chileans completed the online CEER questionnaire, the relationship satisfaction scale (RAS) and dyadic adjustment scale (DAS): 27.8% were male, age *M* = 38.7, *SD* = 10.05, and time of the relationships *M* = 11.27, *SD* = 9.82. The content-based validity study made it possible to determine which items to include in the final version. Two unrelated first-order factors structure of the original test fit (RMSEA = .052, GFI = .97, AGFI = .95; CFI = .99; NFI = .98; and NNFI = .98). The CEER^+^ and CEER^-^ factors presented adequate internal consistency (*α* = .79; ω = .80 and *α* = .85; ω = .85, respectively). The discrimination index of the factors were excellent (CEER^+^ = .55 and CEER^-^ = .63). Validity evidence based on the relations to other variables showed a direct positive relation between CEER^+^, RAS and overall DAS, as well as their factors; and a negative relation between CEER^-^, RAS overall DAS, as well as their factors. The use of this instrument is recommended for the identification of Chilean couples where at least one of the partners has a less favorable opinion of their relationship, providing relevant data for couple’s therapy.

## Introduction

Interpersonal emotion regulation (IER) refers to social interactions aimed at improving or worsening other people’s emotions [1]. It is based on the premise that people not only regulate their own emotions but also seek to influence, affect, or modify other people’s emotional experiences [2]. Niven et al. [3] refer to this as deliberate emotion regulation, the social regulation of affects through strategies such as deliberately attempting to influence the emotional states of others, thus improving, or worsening their affects. This is also known as extrinsic regulation [4]. Studies have shown a direct and significant association between a person’s intention to improve or worsen other people’s emotions and their own well-being or discomfort [5]. In addition, there are hedonic motives related to making others feel good, and the search for individual benefits to making them feel bad [6].

IER is essential to understanding the quality of close bonds such as partner relationships. According to the literature, the way a couple regulates their emotions may affect satisfaction and security with the relationship [7, 8]. In addition, issues associated with regulating emotions have always been associated with severe marital problems, more negative emotional expression, less positive expression, and risk of dissolution [9, 10]. Specifically, the evidence shows that when a person invalidates an expression of emotions by another person (by rejecting or judging them for it), this has negative emotional impacts such as emotional dysregulation [11]. Additional findings show a strong association between IER and positive/close relationships. It is also related to general well-being [12] and the dyadic adjustment, i.e., an adaptation process required for a couple to attain a functional and harmonious relationship as expressed in consensus, satisfaction, cohesion, and affectional expression [13].

Interaction paradigms are commonly used to study affective dynamics in couples. For instance, in video-recorded sessions, dyads discuss stressors, and their emotional responses are recorded [14]. Other approaches include the linguistic analysis of qualitative data such as that included in the Adult Attachment Interview, where trained researchers record the interviews and then code them, generating a conversational protocol that serves as the basis for further analysis [15]; or the use of life diaries [16]. Despite the methodological strengths of these modalities [17], other related phenomena such as extrinsic emotion regulation are not directly observable, and thus require other techniques such as questionnaires [18].

In response to this challenge, the Interpersonal Emotion Regulation Questionnaire (IERQ) was developed by Hofmann et al. [2] to assess the strategies people use to regulate their own emotions through others. Although the psychometric characteristics of this tool are adequate, it does not assess extrinsic regulation. Another instrument, the Brief Emotional Intelligence Scale (BEIS-10) [19], is a five-dimensional scale, also validated in an Italian population [20], which measures an individual’s willingness to explore his/her own emotions and the emotions of others. However, only two of the scale’s dimensions refer to the regulation and evaluation of the emotions of others. These dimensions focus on the individual’s ability to promote positive feelings in others, but not negative feelings or their intentionality, overlooking the fact that both types of emotions coexist in human relationships. Similarly, the Interpersonal Affect Improvement Strategies Questionnaire (IAISQ), developed by López-Pérez et al. [21], focuses on the intention to enhance other people’s emotions, but not on the desire to make the other person feel bad. It presented adequate construct validity evidence and acceptable reliability. Yet another questionnaire, the Intimate Partner Flooding Scale (IPFS), evaluates the effects that one person’s negative emotions have on their partner. Like the others, this instrument does not assess any deliberate intention to make the other person feel bad, although it presents reasonable unifactoriality, excellent internal consistency, and high test-retest reliability [22].

Conversely, the Managing the Emotions of Others Scale (MEOS) allows for a detailed description of the different prosocial and non-prosocial ways in which people manage the emotions of others; however, it does not focus on intimate partners or intentions [23, 24]. Another scale, the Difficulties in Interpersonal Regulation of Emotions (DIRE), designed by Dixon-Gordon et al. [25], relates regulatory difficulties with psychopathology; it does not measure the intention to regulate emotions, though it does present adequate internal coherence and provide evidence of construct and predictive validity. The one scale designed specifically for a Chilean population, the Perceived Emotional Synchrony Scale [26], but it targets people’s perception of synchrony after participating in group activities such as protests.

The Emotion Regulation of Others and Self (EROS) scale, created by Niven et al. [1], is an instrument for assessing individual differences in the strategies used to deliberately regulate emotions in a non-clinical population. It is formed by two subscales: the regulation of one’s own emotions (10 items) and the regulation of other people’s emotions (9 items). In turn, each subscale has two factors showing evidence of construct validity and internal consistency. The EROS was translated into Spanish and validated by Lozano et al. [27], who used it for a sample of elderly Spaniards. Just like the original scale, it had two factors for each subscale. The EROS comprises 24 items on a five-point Likert-type scale (from 1 = not at all, to 5 = a great deal), where 12 items assess the regulation of one’s own positive (ESR^+^) and negative (ESR^-^) emotions, and the other 12 assess the regulation of others’ positive (ERO^+^) and negative (ERO^-^) emotions. Table 1 presents the psychometric properties of instruments that assess interpersonal regulation of emotions, organized according to how closely each is related to extrinsic emotion regulation in couples.

**Table 1:** Instruments that evaluate constructs associated with interpersonal emotion regulation.

Instrument	Authors	Construct definition	Psychometric evidence
Perceived Emotional Synchrony Scale (PESC)	Páez et al. (2015) [26]	The extent to which participants experienced a condition of collective effervescence	Internal consistency α = .94
Interpersonal Emotion Regulation Questionnaire (IERQ)	Hofmann et al. (2016) [2]	A reliance on others in the regulation of one’s own emotions	Internal consistency (α = .89, .91, .94 and. 93, respectively). Good convergent and discriminant validity, with a more modest relationships with other measures of emotion regulation, emotional intelligence, anxiety and depression, and certain coping styles
		Four factors: enhancing positive affect, perspective taking, soothing and social modeling	
Difficulties in Interpersonal Regulation of Emotions (DIRE)	Dixon-Gordon et al. (2018) [25]	Difficulties with intrinsic interpersonal emotion regulation associated with maladaptive patterns (psychiatric symptoms)	Each factor achieved adequate internal consistency (α = .70 –. 87), construct and predictive validity evidence
		Two factors: interpersonal strategies (venting and reassurance-seeking subscales); and intrapersonal strategies (acceptance and avoidance subscales)	
Intimate Partner Flooding Scale (IPFS)	Foran et al. (2018) [22]	The effects of one partner’s anger—unexpected/intense, overwhelming, and disorganizing (i.e. flooding) on the other	Internal consistency for women (α = .96) and men (α = .96). Test–retest .66 for men and .79 for women. Appropriate construct validity evidence
Brief Emotional Intelligence Scale (BEIS-10)	Davies et al. (2010) [19]	Individual willingness to exploring one’s and others’ emotions (with the aim of fostering positive emotions).	Reliability (α .48, .35, .40, .41) and .40 respectively). Content and construct validity evidence.
		Five factors: appraisal of own emotions, appraisal of others’ emotions, regulation of own emotions, regulation of others’ emotions, and utilization of emotions	Italian version (Durosini et al., 2020) [20]. Internal consistency (α = .73) and item-total correlation analysis. CFA analysis yielded good fit indices for the five-factor structure
Interpersonal Affect Improvement Strategies Questionnaire (IAISQ)	López-Pérez et al. (2019) [21]	The specific strategies people may use to improve others’ mood.	Reliability for positive engagement ω = .78, and for acceptance ω = .74. Both factors were positively related to extraversion and agreeableness
		Two factors: positive engagement and acceptance	
Managing the Emotions of Others Scale (MEOS)	Austin et al. (2013) [23]	Provide coverage of the different ways (prosocial and non-prosocial) in which people manage the emotions of others.	Internal reliabilities were .91, .88, .82, .85, .68, .81, respectively. Range of test–retest r = .71–.83. Strong correlations factor scores with short measures of the Big Five, the Dark Triad and trait emotional intelligence.
		Six factors: mood enhancing, mood worsening, concealing emotions from others, use of inauthentic displays, poor emotion skills, and use of diversion to enhance another’s mood	Polish version (Jankowsky et al., 2016) [24]. Mood enhance is strongly related to performance-based emotional intelligence.
			Among the Dark Triad, Narcissism was related to the greatest number of MEOS subscales—all except poor emotional skills. MEOS has a similar factor structure, reliability, and pattern of correlations with personality and emotional intelligence
Emotion Regulation of Others and Self (EROS) scale	Niven et al. (2011) [1]	To assess individual differences in the strategies used to deliberately regulate emotions (in a non-clinical population)	Each subscale shows evidence of construct validity and internal consistency (*α* .87, .83, .90 and .67 respectively). EROS was translated into Spanish and validated by Lozano et al. (2015) [27], who used it for a sample of elderly Spaniards. It obtained evidence of construct validity and good internal consistency (*α* .86, .66, .76 and .83 respectively)
		Four factors: intrinsic affect-improving, intrinsic affect worsening, extrinsic affect-improving and extrinsic affect-worsening	

The instruments are ordered based on how closely they fit the definition of the measured constructs in the new questionnaire (the first is the poorest fit and the last, the best fit).

In summary, to gauge extrinsic emotion regulation, scholars adapted and validated these instruments for close interpersonal relationships in general, but not specifically for couple relationships, and in countries other than Chile [27]. There are currently no instruments to assess extrinsic emotion regulation in non-pathological couples in any country. For these reasons, we considered fast, valid, and reliable self-reporting measures were necessary to assess the social interdependence used to configure and improve couple relationships [28, 29]. In this sense, the present study deepens the understanding of such interdependence and how it impacts a couple’s regulation of their emotional life [30, 31].

This article describes the adaptation of the “Others” subscale (positive and negative) from the Emotion Regulation of Others and Self (EROS) instrument for couples in Chile. Specifically, it examines validity evidence based on the adapted test content, its internal structure, and reliability. In addition, validity evidence is presented drawing on the association with other variables and criteria measures expected to predict the test’s outcome [32, 33]. Specifically, the extrinsic regulation of positive emotion is expected to be associated with increased satisfaction with one’s relationship and greater dyadic adjustment [8, 34]. Conversely, in the case of extrinsic regulation of negative emotion, the opposite outcome is expected.

## Method

### Participants

Based on the instrument’s content, forty Spanish speakers were chosen for the validity study. All who met the following inclusion criteria were contacted about assessing the CEER questionnaire: a bachelor’s degree or degree in psychology with experience in instrument design, and clinical experience in couples, or the field of emotion regulation. Twenty-three participants (57%) ultimately participated in the study. This percentage represented a moderate number of experts to assess a systematized procedure for a validity study based on test content (10 < N < 30) [35]. Twenty participants (87%) were Chilean nationals and three (23%) had been residents of Chile for at least five years. Their average age was *M* = 44 years old (*SD* = 7.09). Thirteen (56.5%) were men and 10 (43.5%) were women. In relation to professional experience, 11 (47.8%) worked in fields related to social and health psychology and the psychology of emotions, four (17.4%) in couples’ therapy, and eight (34.8%) in social science methodologies. In terms of academic degrees, 10 (43.5%) hold a Ph.D., 12 (52.2%) a master’s degree, and one (4.3%) a bachelor’s degree.

In accordance with the instrument’s internal structure, the reliability test, and validity evidence based on relations to other variables, a total of 528 people were chosen for the sample. All met the following inclusion criteria and participated in the study: (a) age ≥ 18 years, (b) were either Chilean citizens or residents (years ≥ 5), (c) were in a relationship of ≥ 6 months at the time of filling out the questionnaires, (d) had a high school degree. Participants ranged in age from 18 to 74, with an average *M* = 38.7 (*SD* = 10.05); the amount of time of their relationships ranged from one to 55 years, *M* = 11.27 (*SD* = 9.82); 147 (27.8%) were male and 381 (72.2%) were female; 503 (95.3%) declared to have a heterosexual, 14 (2.7%) homosexual, and 11 (2%) bisexual orientation; regarding educational attainment, 1 (0.2%) reported primary education, 21 (4%) high school, 426 (80.6%) technical training or university, and 80 (15.2%) graduate school; 392 (74.2%) lived with their partner, and 136 (25.8%) did not.

### Instruments

The 12-item Spanish version of the Emotion Regulation of Others subscale (EROS) [27] was used to obtain validity evidence, based on the test content, and assessed by experts (available at https://forms.gle/bHneijX4mvB7rp7y5). These items, originally designed for any close relationship, were adapted to couple relationships, replacing the term “someone” with “my partner” in all cases. Furthermore, with the assistance of a language specialist, the survey was localized for its application in a Chilean sample, replacing some of the words used in Spain with their Chilean equivalents. Thus, a cultural and functional (rather than literal) equivalence was achieved [36, 37]. Specifically, “I have pointed out (*he señalado*)” was changed to “I said (*hablé*)”; the Spanish term for weak points, “*puntos flacos*” was changed to “*puntos débiles*,” which is more common in Chile (item 3); “I explained (*expliqué*)” was changed to “I expressed (*expresé*)” (item 4); “I discussed his/her positive characteristics (*comenté sus características positivas*)” was changed to “I spoke about his/her qualities (*le hablé de sus cualidades*)” (item 5); “unpleasant (*antipático*)” was changed to “rude (*pesado*)” (item 8), and “I reproached (*recriminé*)” was changed to “I complained (*reclamé*)” (item 10). Finally, item four was simplified by eliminating “me or others (*a mi o a otros*)” and “for having done so (*por haberlo hecho*).”

Two factors (made up of the same items from the original scale) were considered: (1) the intent of the Positive Couples Extrinsic Emotion Regulation, CEER^+^ (makes him/her feel good or better) with six items, and (2) the intent of Negative Couples Extrinsic Emotion Regulation, CEER^-^ (makes him/her feel bad or worse) with six items. Each item was assessed as essential, adequate, or inadequate. A section was provided for recommendations on improving the scale.

The Couples Extrinsic Emotion Regulation (CEER) questionnaire is the instrument that resulted from the validity study based on the instrument’s content. It is comprised of the previously two mentioned subscales (CEER^+^ and CEER^-^), each with six items, on a five-point Likert scale (1 = never to 5 = always).

Hendrick’s Relationship Assessment Scale (RAS): This unifactor scale assesses a person’s overall satisfaction with the relationship, with a high predictive capacity for which relationships will last (91%) and which will end (86%) [38]. A self-administered survey, it is comprised of seven items that must be ranked on a five-point Likert scale (1 = low satisfaction, to 5 = high satisfaction). It showed excellent reliability indicators for the Chilean population (*α* = .92) [39].

Spanier’s Dyadic Adjustment Scale (DAS) [40]: This self-administered instrument is widely used to assess the quality and adjustment of couples [41]. With 32 items (one item on a seven-point Likert scale, 27 on a six-point Likert scale, two on a five-point scale, and two on a dichotomous scale), it yields an overall score along with a score for each of its four subscales (consensus, satisfaction, cohesion, and affectional expression). The Spanish version of the DAS had the same internal structure as the original scale [42], along with appropriate reliability (*α* = .80 and *α* = .92) [43, 44].

### Procedure

The Research Ethics Committee at Diego Portales University (Chile) approved the study (approval number 012–2019). All participants provided written informed consent.

In order to perform the validity study based on the instrument’s content, a link to the questionnaire (Google Forms) was sent by email, after contacting most of the experts to explain the purpose of their involvement; the study was presented as voluntary and non-anonymous. Answers were obtained using a spreadsheet generated on Google Drive.

In order to perform the study of the validity evidence based on the instrument’s internal structure and relations to other variables, non-probabilistic sampling was used, and 4,380 individuals were recruited via email using two combined procedures: (a) incidental sampling: information taken from distribution lists accessed by the authors (from the university, professional training, the parents of pediatric patients, insurance companies, cultural and sports associations, and schools); and (b) snowball sampling: given that the Chilean population tends to be reluctant to talk about their private lives, some participants from the incidental sampling recommended other potential participants from their respective social networks. The final sample consisted of 528 participants, 12.1% of the total contacted, who anonymously responded to three online Google Form questionnaires (CEER, RAS, DAS). It should be noted that the digital questionnaire was configured so that answers for each item were mandatory in order to avoid missing data. Eight duplicates were eliminated. It is useful to note that some studies have supported the psychometric equivalence between the paper-and-pencil and online questionnaires used for intimate partner research [45].

### Data analysis

Excel (Microsoft Corp., Chicago, IL, 2018), SPSS 23 (IBM Corp., Armonk, NY, 2015), PRELIS and LISREL 9.3 (Scientific Software International Inc., Lincolnwood, IL, 2017), JASP (JASP Team, Amsterdam, The Netherlands, 2020) and FACTOR (Ferrando & Lorenzo-Seva, Tarragona, Spain, 2017) were the software used for data recording and analysis.

The content validity calculation (CVC) was performed with Lawshe’s coefficient [46]:

$$CVC=\frac{n-\frac{N}{2}}{\frac{N}{2}}$$

where *n* is the number of experts who deemed the item essential and *N* is the number of experts who evaluated the item. The CVC ranges from +1 to -1, with positive scores indicating adequate validity based on content. A minimum CVC of .42 for 20 experts (items with lower values would have been eliminated) is suggested [46].

In order to obtain validity evidence based on the internal structure of the instrument, Mardia’s multivariate skewness (S) and kurtosis (K) were tested [47] first, considering the items as continuous variables. When the significance of S or K was lower than .05, multivariate normality assumption was not met. In the case of an asymmetric distribution or excess kurtosis of ordinal items, the use of polychoric correlations was advised [48, 49]. The bivariate normality assumption needed to be fulfilled to use the polychoric correlation matrix [50]. This assumption was checked calculating Chi-square (*X*^*2*^) for each pair of correlations and the Bonferroni correction was used to calculate the number of times the null hypothesis was rejected. Thus, statistical significance was compared with the result obtained by applying the formula *α*/c, where *α* = .05, corresponded to a 95% confidence level and c was the number of contrasts (c = [number of items x number of items—1] / 2) [51]. Since *X*^*2*^ is very sensitive for large samples, it is often used to indicate a relative fit rather than an absolute fit. For this reason, the root mean square error of approximation (RMSEA) was also calculated [52]. When the RMSEA for each polychoric correlation was less than .1, the estimation of the parameter was not affected. Then, polychoric correlations matrix [53, 54] and the asymptotic variance-covariance matrix were estimated. The Diagonally Weighted Least Squares (DWLS) method was used for the estimates [54].

In order to assess data dimensionality, a parallel analysis based on minimum rank factor analysis was carried out [55]. Additionally, at the item-level, the overall assessment indexes were a) Unidimensional Congruence (UniCo), b) Explained Common Variance (ECV), and c) Mean of Item Residual Absolute Loadings (MIREAL) [56]. Data can be treated as essentially unidimensional when UniCo is above .95, ECV is above .85, and MIREAL is above .30.

**Fig 1:**
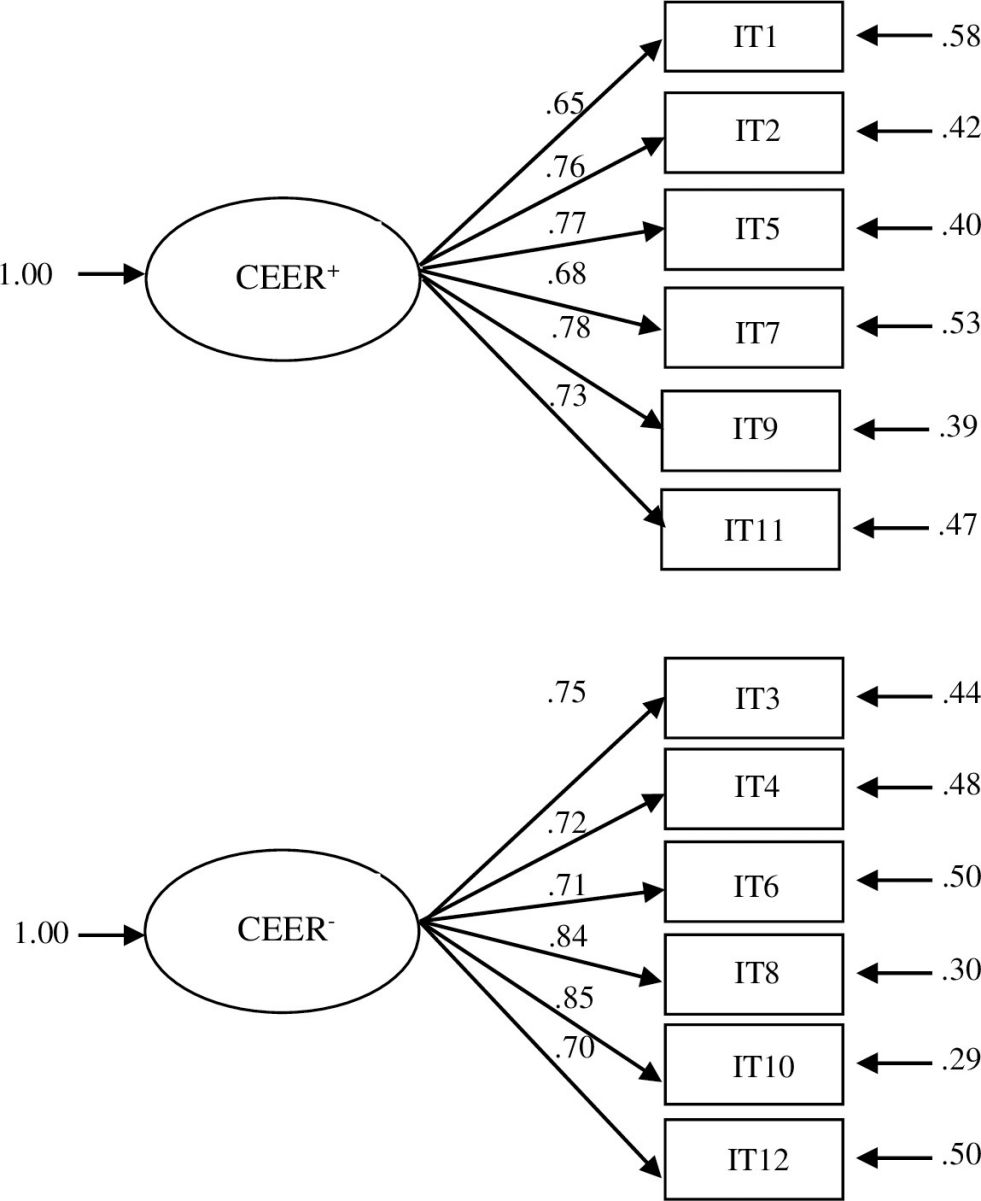
Standardized factor loadings for the CEER questionnaire model of two unrelated first-order factors (CEER^+^ = positive Couples Extrinsic Emotion Regulation and CEER^-^ = negative Couples Extrinsic Emotion Regulation).

According to the previous dimensionality analysis and the second objective of the study, a confirmatory factor analysis (CFA) was carried out to study the level of fitness of our data with the model in the original test [27]. It was a first-order structure with two unrelated factors, each with six items (see Fig 1).

The model’s adjustment was assessed using different fit indexes [57]: (a) *X*^*2*^, accepting the null hypothesis as a good model fit (*p* ≥ .05); (b) the consistent Akaike information criterion (CAIC) stating that a good fit is when the index value was closer to the value of the saturated model instead of the independent one (low values) (c) the RMSEA, where values between .05 and .08 were considered a reasonable fit [58]; (d) the goodness-of-fit index (GFI) and adjusted GFI (AGFI); (e) the comparative fit index (CFI) [59], (f) the normalized fit index (NFI); and (g) the non-standardized NFI fit index (NNFI). Values above .9 were considered adequate for (d) to (g).

Once the factor structure of the model was tested, the internal consistency was ascertained using Cronbach’s α [60], George and Mallery’s criteria [61] and McDonald’s ω [62]. Values above .90 were considered excellent; between .80 - .90, good; and between .70 - .80, acceptable. The average discrimination index was assessed based on Linnette’s criteria [63], with values greater than .35 considered excellent.

The following item descriptive statistics and metric indexes by factor were presented in the CEER questionnaire: skewness (S), kurtosis (K), item discrimination (ID), Cronbach’s alpha if the item was deleted (α); McDonald’s omega if the item was deleted (ω); and reliability index (RI).

Before assessing validity based on the relation to other variables (satisfaction with the relationship and dyadic adjustment), normality assumptions were verified with the Kolmogorov-Smirnov test (normal distribution assumed *p* > .05); linearity was checked (met when *p* < .05); and error independence were verified with the Durbin-Watson test (values between 1.5 < *d* < 2.5 were considered adequate). Since not all assumptions were satisfactory, Spearman’s bivariate correlations (*ρ*) were calculated and interpreted following Cohen’s criteria [64] with values close to .1, .25, and .4 (absolute value) reflecting a low, moderate, and high effect magnitude, respectively. In order to estimate the true relationship between the test and the criterion variables, a structural equation model (SEM) was analyzed. *X*^*2*^ with *p* > .05 was interpreted as model fitness; and Student t was used to study the significance of each parameter (with a confidence level of 99%, values over 2.58 were considered significant).

## Results

### Validity study based on the instrument’s content

In relation to this study, raw data are available in S1 and S2 Datasets. In the first assessment (phase 1), of the 12 items, three (items 2, 4, and 5) met the inclusion criteria (see Table 2). Suggestions for improvement were collected for all items that failed to meet these criteria (four were assigned a negative value, and five, a value between 0 and .3).

**Table 2:** Validity results based on test content.

Items in their final Spanish version [with English translation]	CVC Phases	
	1^st^	2^nd^
1. En momentos difíciles apoyé a mi pareja con un consejo con la intención de que se sintiera mejor [During difficult times, I supported my partner, giving advice with the intention of making him/her feel better]	**-.24**	1
2. Hice algo agradable junto a mi pareja con la intención de que se sintiera mejor [I did something nice with my partner with the intention of making him/her feel better]	.57	1
3. Le hablé a mi pareja de sus defectos con la intención de que se molestara [I told my partner about his/her shortcomings with the intention of upsetting him/her]	**.22**	.91
4. Le expresé a mi pareja cuánto me había hecho sufrir con la intención de que se sintiera mal [I expressed to my partner how much he/she had made me suffer with the intention of making him/her feel bad]	.65	1
5. Le hablé a mi pareja de sus cualidades positivas con la intención de que se sintiera mejor [I told my partner about his/her positive qualities with the intention of making him/her feel better]	.74	1
6. Fingí enojarme con mi pareja con la intención de que se sintiera mal [I pretended to be angry with my partner with the intention of making him/her feel bad]	**.30**	1
7. Escuché los problemas de mi pareja con la intención de que se sintiera mejor [I listened to my partner’s problems with the intention of making him/her feel better]	**.22**	.74
8. Fui desagradable con mi pareja con la intención de que se sintiera mal [I was disagreeable with my partner with the intention of making him/her feel bad]	**.04**	.83
9. Hice reír a mi pareja con la intención de que se sintiera mejor [I made my partner laugh with the intention of making him/her feel better]	**-.04**	.83
10. Critiqué el comportamiento de mi pareja con la intención de que se sintiera mal [I criticized my partner’s behavior with the intention of making him/her feel bad]	**-.22**	.83
11. Dediqué más tiempo a mi pareja para intentar que se sintiera mejor [I spent more time with my partner with the intention of making him/her feel better]	**-.04**	.83
12. No le hice caso a mi pareja con la intención de que se sintiera mal [I did not listen to my partner with the intention of making him/her feel bad]	**.22**	.91

CVC = Content validity calculation. Values that did not meet the inclusion criteria are shown in bold (CVC < .42).

All the suggested adjustments were made. Four people proposed changing the adjective “worse (*peor*)” to “bad (*mal*)” (items 4, 6, 8, 10, and 12), to reduce the callousness of the statement for Chileans, who are particularly sensitive to words with strong negative connotations. For the same reason, in item 3 “to make him/her feel worse (*se sintiera peor*)” was replaced by “to upset him/her (*se molestara*).” The remaining modifications were each suggested by one person. “To do (*para hacer*),” “to (*para*),” “to try (*para intentar*),” and “to achieve (*para conseguir*)” were replaced by “in an attempt to (*con la intención de*)” in items where this phrase was not originally included (items 3, 4, 5, 6, 7, 8, and 10). The purpose of these changes was to emphasize the intention behind the action and respond to existing semantic and cultural nuances between Chile and Spain. The remaining modifications involved the substitution of synonyms, sometimes in relation to nuanced meanings and in other cases, grammatical structures: “I have given him/her useful advice (*le he dado un consejo útil*)” was changed to “during difficult times, I supported my partner, giving advice (*en momentos difíciles apoyé a mi pareja con un consejo*)” (item 1); “I did something nice with … (*hice algo agradable con* …)” to “I did something nice together with … (*hice algo agradable junto a* …” (item 2); “weak points (*puntos débiles*)” to “flaws (*defectos*)” (item 3); “how he/she had made me suffer (*cómo me había hecho sufrir*)” to “how much he/she had made me suffer (*cuánto me había hecho sufrir*)” (item 4); “rude (*pesado*)” to “disagreeable (*desagradable*)” (item 8); I complained (*reclamé*)” to “I criticized (*critiqué*) (item 10); and “I did not (*no hice*)” was replaced by “I ignored (*no le hice caso*)” in item 12. For some items, words were added: “positive (*positivas*)” in item 5; “more (*más*)” was added before “time (*tiempo*)” in item 11; and “my partner (*mi pareja*)” was added.

Table 2 shows the items in their final version, after all amendments were incorporated. The CVC of the items after their modification (phase 2) ranged from .74 to 1, with all exceeding the inclusion criteria.

### Validity evidence based on the instrument’s internal structure

In relation to the CEER questionnaire, the model test of two unrelated first-order factors, internal consistency, discrimination, descriptive and metric properties of items by factor, and validity study based on the relations to other variables, the raw data are available in S3 and S4 Datasets.

Multivariate skewness was met, S(364) = 3079.29 (p > .05), but multivariate kurtosis was not met, K = 46.49 (p < .001). As a result, we concluded that multivariate normality was not met.

After rejecting multivariate normality, a bivariate normality assumption was tested in order to be able to use a polychoric correlations matrix. A total of 66 contrasts were obtained (12 x 11/2). The premise of bivariate normality was accepted as the statistical significance associated with *X*^*2*^ was greater than .05 / 66 = .0007 on 59 occasions (89.4%). Additionally, all RMSEA values were below .1.

With regard to the analysis of dimensionality, the parallel analysis based on minimum rank factor advised for two dimensions. The following indexes suggested that the data should not be treated as essentially unidimensional: UniCo = .850, 95% CI [.744, .905]; ECV = .634, 95% CI [.567, .688]; and MIREAL = .466, 95% CI [.434, .503].

The CFA with the two unrelated first-order factors structure fit. There was no adjustment with *X*^*2*^(54) = 161.48, *p* < .001, possibly due to the large sample size. The other indexes showed an adequate fit of the proposed model as follows: CAIC = 304.02 (saturated CAIC = 566.99; independent CAIC = 5351.69); RMSEA = .052, 90% CI [.040, .063]; GFI = .97; AGFI = .95; CFI = .99; NFI = .98; and NNFI = .98. The model obtained appropriate *λ*, with values ranging from .65 to .85 (see Fig 1).

### Instrument’s internal consistency, average discrimination index and items analysis

The CEER^+^ and CEER^-^ factors presented good internal consistency (*α* = .79; ω = .80 and *α* = .85; ω = .85, respectively). The factors discrimination indexes were excellent (CEER^+^ = .55 and CEER^-^ = .63). Table 3 introduces item descriptive statistics. All items presented negative S, ranging from -.81 to -2.33, and all but one obtained positive K, ranging from -.27 to 7.07, with excellent ID (.43, .71), good internal consistency if the items were deleted (α: .74 - .84; ω: .76 - .84) and adequate RI (.23, .74).

**Table 3:** Item descriptive statistics and metric indexes by factor in the CEER questionnaire.

Factor	Items	M	SD	S	K	ID	α	Ω	RI
CEER^+^	IT1	4.670	0.528	-1.377	1.402	0.433	0.787	0.792	0.229
	IT2	4.472	0.671	-1.085	0.734	0.577	0.755	0.770	0.387
	IT5	4.445	0.745	-1.373	1.965	0.619	0.744	0.755	0.461
	IT7	4.595	0.712	-2.330	7.071	0.476	0.778	0.790	0.339
	IT9	4.489	0.747	-1.566	2.549	0.607	0.747	0.756	0.453
	IT11	4.218	0.856	-1.126	1.445	0.583	0.755	0.765	0.499
CEER^-^	IT3	4.125	0.968	-0.996	0.413	0.622	0.826	0.831	0.602
	IT4	3.966	1.100	-0.809	-0.272	0.606	0.831	0.836	0.667
	IT6	4.288	0.980	-1.319	1.029	0.595	0.831	0.836	0.583
	IT8	4.163	1.003	-1.022	0.186	0.710	0.809	0.814	0.712
	IT10	4.100	1.038	-1.009	0.272	0.709	0.809	0.812	0.736
	IT12	4.403	0.872	-1.415	1.360	0.560	0.838	0.839	0.488

CEER^+^ = Positive Couples Extrinsic Emotion Regulation; CEER^-^ = Negative Couples Extrinsic Emotion Regulation; S = skewness; K = kurtosis; ID = item discrimination; α = Cronbach’s alpha if the item is deleted; ω = McDonald’s omega if the item is deleted; RI = reliability index.

### Validity evidence based on relations to other variables (test-criteria association)

None of the variables met the assumption of normality, with *p* ≤ .001. However, all associations met the assumptions of linearity (with *p* < .001) and error independence (1.825 < *d* < 2.057).

The validity evidence based on the relations of both CEER factors was calculated using the following criteria measures: RAS, DAS, and their respective factors (see Table 4). The reported measures also showed good consistency for RAS (*α* = .9; ω = .9), and for overall DAS (*α* = .89; ω = .9). In terms of the consensus and satisfaction subscales, results in general were adequate (*α* = .87; ω = .87 and *α* = .43; ω = .62 respectively); for the cohesion and affect expression subscales, they were acceptable (*α* = .67; ω = .68 and *α* = .68; ω = .75 respectively).

**Table 4:** Bivariate correlations as validity evidence based on the relation of the instrument to other variables (test—criteria correlation).

*Measures*	1	2	3	4	5	6	7	8
1. CEER^+^	-							
2. CEER^-^	-.18*	-						
3. RAS	.37*	-.37*	-					
4. Consensus (DAS)	.41*	-.30*	.70*	-				
5. Satisfac. (DAS)	.41*	-.41*	.80*	.66*	-			
6. Cohesion (DAS)	.41*	-.25*	.51*	.55*	.54*	-		
7. A. expres. (DAS)	.34*	-.33*	.60*	.55*	.56*	.54*	-	
8. Global DAS	.47*	-.38*	.78*	.89*	.87*	.75*	.70*	-
	*Descriptive statistics*						
*Possible range*	[1, 5]	[1, 5]	[1, 5]	[0, 65]	[0, 50]	[0, 24]	[0, 12]	[0, 151]
*M*	4.48	1.83	4.20	51.23	40.63	17.76	11.07	120.69
*SD*	0.50	0.75	0.73	8.34	7.21	3.94	2.48	18.67

*N* = 528. Spearman correlations are shown. CEER^+^ = Positive Couples Extrinsic Emotion Regulation; CEER^-^ = Negative Couples Extrinsic Emotion Regulation; RAS = Relationship Assessment Scale; DAS = Dyadic Adjustment Scale; Satisfac. = Satisfaction; A. expres. = Affectional expression. *p < .01.

Among all the variables mentioned, there were bivariate correlations with *p* < .01 in the expected sense: the higher the CEER^+^, the higher the RAS (*ρ* = .37) and overall DAS (*ρ* = .47), as well as their factors; and the higher the CEER^-^, the lower the RAS (*ρ* = -.37) and overall DAS (*ρ* = -.38), as well as their factors. In magnitude terms, CEER^+^ as well as CEER^-^ correlations and the criterion variables were high to moderate. The correlation between both CEER factors was statistically significant, although with a low-medium magnitude (*ρ* = -.18).

**Fig 2:**
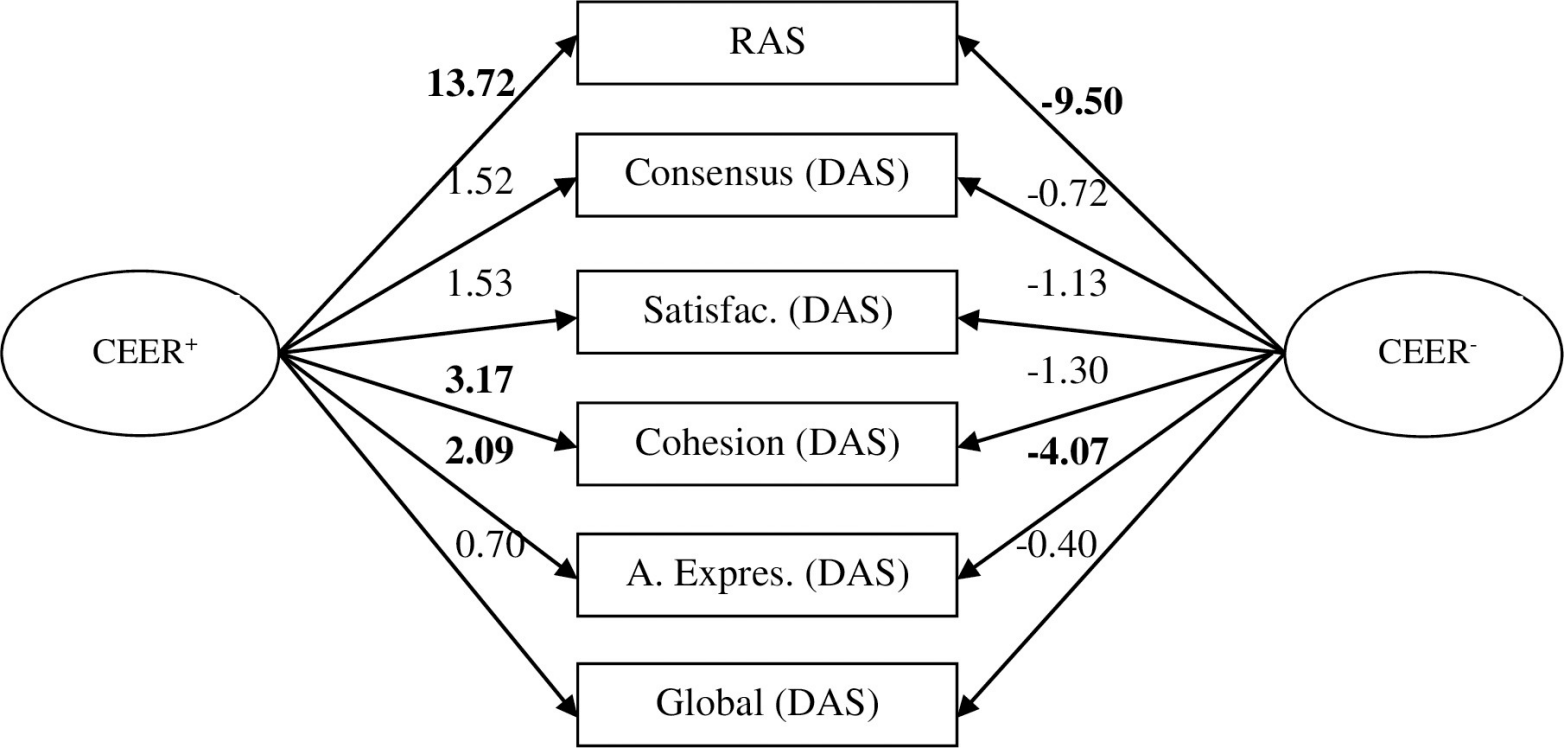
Parameters significance of the effects of X on Y based on SEM (CEER^+^ = positive Couples Extrinsic Emotion Regulation; CEER^-^ = Negative Couples Extrinsic Emotion Regulation; RAS = Relationship Assessment Scale; DAS = Dyadic Adjustment Scale; Satisfac. = Satisfaction; A. expres. = Affectional expression. Significant results -Student t values over 2.58 with a confidence level of 99%- are marked in bold).

SEM tested was formed by two predictors, i.e., CEER^+^ (X_1_) and CEER^-^ (X_2_), on six criteria, i.e., RAS (Y_1_), Consensus–DAS–(Y_2_), Satisfaction–DAS–(Y_3_), Cohesion–DAS–(Y_4_), Affectional expression–DAS–(Y_5_) and Global DAS (Y_6_). The data fitted the model, *X*^*2*^(16) = .14, *p* > .05. Standardized effects and parameters significance of the test are presented in Table 5. Additionally, parameters significance is illustrated in Fig 2.

**Table 5:** Standardized effects and parameters significance (structural equation model).

	CEER+		CEER-	
	Λ	T	λ	T
RAS	.36*	13.72	-.29*	-9.50
Consensus (DAS)	.34	1.52	-.24	-.72
Satisfac. (DAS)	.30	1.53	-.32	-1.13
Cohesion (DAS)	.37*	3.17	-.18	-1.30
A. expres. (DAS)	.32*	2.09	-.26*	-4.07
Global DAS	.39	.70	-.30	-.40

CEER^+^ = Positive Couples Extrinsic Emotion Regulation; CEER^-^ = Negative Couples Extrinsic Emotion Regulation; RAS = Relationship Assessment Scale; DAS = Dyadic Adjustment Scale; Satisfac. = Satisfaction; A. expres. = Affectional expression; λ = standardized factor loadings; t = Student t (values over 2.58 are significant with a confidence level of 99%). *p < .01.

The model showed a direct positive relation between CEER^+^, RAS and overall DAS, as well as their factors; it revealed a negative relation between CEER^-^, RAS and overall DAS, as well as their factors. However, seven out of the twelve parameters did not yield statically significant values.

## Discussion

The CEER questionnaire showed validity evidence based on the test content after its items were adapted from the original EROS [27] to Chilean couples. The existence of two independent factors was confirmed (CEER^+^ and CEER^-^). Both factors have adequate reliability and validity evidence based on the internal structure, in keeping with the results obtained when the instrument was initially used (for the general population) and validated with a Spanish sample [27]. Both scale factors correlated with other variables as hypothesized [8, 10, 13, 34]. This outcome provided some validity evidence based on the test-criteria association for the instrument.

By focusing on the IER’s deliberate intention and not only on the strategies they use, the CEER questionnaire allowed for the gathering of valuable data not always observable in studies about the emotional dynamics of romantic dyads [4, 5]. This could facilitate the identification of couples where at least one of the partners has a less favorable opinion of the relationship, providing relevant data for couple’s therapy. For example, as part of integrative behavioral couples’ therapy (IBCT), this tool could detect behavior considered problematic or harmful for the couple and usually contextualized [65] to determine the relationship differences and use acceptance or tolerance strategies to work through them.

It is important to emphasize that a validation process is a continuum in which evidence continually accumulates (content, construct, appearance, consequences, criteria, utility, etc.). The way in which the scores behave under other relevant conditions, though not examined in this work, will be important for future research. Studies could focus, for example, on couples at different stages of the relationship (newlyweds versus the most stable over time) and whether or not they cohabit. Another question is whether the intention underlying extrinsic regulation in a couple can be mediated by the other’s perception of this intention, since knowledge is limited to the behavioral level [66].

Some relevant limitations to the study include the use of non-probabilistic sampling (which could have generated biased data) and the low diversity of some characteristics (e.g., sexual orientation, as this was mainly a heterosexual sample; or language and location, i.e., a Spanish-speaking sample limited to Chile). Therefore, we suggest extrapolating the results with caution. In future research, a randomized procedure would be advisable, as would greater heterogeneity in terms of both the socio-demographic characteristics of the sample and the assessment of the relationship itself. The integration of these components would allow for an invariance study to determine whether the outcomes from this study are like those of different population groups. Couples who present clinical cases of abuse and domestic violence would be a suitable target population in future research, using the CEER questionnaire as another precise intervention tools along with other tests such as the Scale of Psychological Abuse in Intimate Partner Violence (EAPA) [67]. The CEER questionnaire could thus serve as an additional resource in a psychopathological diagnosis and the increase of the methodological quality of the interventions [68].

## Supporting information

S1 Dataset Raw data gathered in validity study based on the instrument’s content (English version). (XLSX)

S2 Dataset Raw data gathered in validity study based on the instrument’s content (Spanish version). (XLSX)

S3 Dataset Raw data gathered in validity study based on the instrument’s internal structure, internal consistency, average discrimination index, item analysis, and validity evidence based on the relations to other variables (English version). (XLSX)

S4 Dataset Raw data gathered in validity study based on the instrument’s internal structure, internal consistency, average discrimination index, item analysis, and validity evidence based on the relations to other variable (Spanish version). (XLSX)
